# Neutrophil extracellular traps and pulmonary fibrosis: an update

**DOI:** 10.1186/s12950-023-00329-y

**Published:** 2023-01-19

**Authors:** Suyan Yan, Meiqi Li, Baocheng Liu, Zhenzhen Ma, Qingrui Yang

**Affiliations:** Department of Rheumatology and Immunology, Shandong Provincial Hospital Affiliated to Shandong First Medical University, No. 324, Jingwu Road, Huaiyin District, Jinan, 250021 Shandong China

**Keywords:** Pulmonary fibrosis, Neutrophil extracellular traps, Interstitial lung disease

## Abstract

Pulmonary fibrosis (PF) is a serious and often fatal illness that occurs in various clinical settings and represents a significant unmet medical need. Increasing evidence indicates that neutrophil extracellular traps (NETs) contribute significantly to the progression of PF. Therefore, understanding the pathways by which NETs contribute to the disease is crucial for developing effective treatments. This review focuses on the formation of NETs and the common mechanisms of NETs in PF.

## Highlights

1. This review focuses on NETs formation and the role of NETs in PF and presents research progress.

2. We describe specific mechanisms by which NETs induce PF in fibroblasts while revealing the transduction mechanisms that remain to be investigated.

3. We explore the possible consequences of NETs action on lung epithelial cells, including NETs-induced lung epithelial damage and NETs-driven epithelial mesenchymal transition.

4. We introduce recent key findings about the role of NETs-mediated chronic inflammation in PF and the effect of autophagy-driven production of NETs in fibrosis.

## Introduction

Neutrophil extracellular traps (NETs), a meshwork structure that captures bacteria, fungi and viruses, were discovered by Volker Brinkmann in 2004 [1]. DNA, histones, and antimicrobial proteins are found in NETs, which are produced by activated neutrophils, and high concentrations of NETs around pathogens are considered antibacterial. NETs play an important role in host defense mechanisms and in non-infectious diseases, such as autoimmune diseases, vasculitis, psoriasis and other pathological processes, including coagulation, thrombosis [2, 3], diabetes mellitus [4], atherosclerosis [5, 6], cystic fibrosis [7] and malignant tumors [8, 9]. Furthermore, elevated levels of NETs have been detected in tissues of patients with PF, and they are associated with disease progression [10]. Evidence indicates that NETs components such as DNA, myeloperoxidase (MPO), neutrophil elastase (NE) and histones release cytokines that lead to inflammation, epithelial-mesenchymal transformation (EMT) or epithelial damage, all of which promote the progression of lung fibrosis. In this review, we discuss the formation of NETs, their role in PF and current advances in studying NETs in PF.

### Role and formation of NETs

As the body’s primary defense cells, neutrophils enhance the immune response by phagocytosis, degranulation and release of reactive oxygen species (ROS) [11–13]. NETs are extracellular structures composed of chromatin and granule proteins that bind and kill microorganisms. NETosis is the process of cell death following neutrophil activation, which differs from apoptosis and necrosis in morphological and molecular features. This novel form of cell death involves the formation of NETs and then continuing to play an important role in the immune response [1, 14].

Research has demonstrated that activated NADPH oxidase may produce ROS, and the main pathways that activate NADPH oxidase include PKC-Raf, MERK/ERK and PI3K/Akt [15–17]. In response to the generated ROS, MPO is stimulated, and elastase is activated and migrates from asplenophilic granules to the nucleus by the synergistic action of ROS and MPO [18, 19]. As NETs form, NE digests nucleosomal histones and promotes extensive chromatin deglycosylation, late binding of MPO to chromatin stimulates NE to depolymerize chromatin, and granulins bind to depolymerized chromatin released by neutrophils [20]. Notably, a portion of the formed NETs is mediated by mitochondrial ROS without NADPH oxidase activity [21, 22]. Peptidylarginine deiminase type 4 (PAD4) is involved in developing NETs because PAD4-Knock out (KO) mice were found to eliminate NETs and induce histone guanylation, and this increase in histone guanylation was associated with chromatin deprotonation during NETs formation [23]. By converting histone arginine to citrulline, PAD4 reduces the strong positive charge of histones, thus weakening histone DNA binding. This weakened interaction causes nucleosome unpacking and chromatin depolymerization [24]. Interestingly, according to a recent study, the formation of NETs is independent of ROS and may result from the multitude of molecules released by *Staphylococcus aureus* during neutrophil cleavage [25].

### Interstitial lung disease

Interstitial lung disease (ILD), also called diffuse parenchymal lung disease, is a group of disease with diffuse lung parenchymal, alveolar inflammation and interstitial fibrosis as basic pathological changes [26]. ILD is etiologically complex and is associated with more than 200 diseases, which can be categorized according to known etiology (such as environmental exposure, drug toxicity and connective tissue disease) or unknown etiology (such as idiopathic pulmonary fibrosis (IPF)) [27]. IPF, the most common and aggressive form of ILD, is characterized by the imaging and pathologic features of conventional interstitial pneumonia without an established etiology or correlation with PF-related diseases [28]. According to current estimates, IPF prevalence ranges from 0.57 to 4.51 per 10,000 people in Asia-Pacific countries, 0.33 to 2.51 in Europe, and 2.40 to 2.98 in North America [29]. ILD is also a common pulmonary complication in patients with connective tissue diseases (CTDs). Various CTDs, such as rheumatoid arthritis, systemic sclerosis (SSc), polymyositis and dermatomyositis, Sjögren’s syndrome (SS), systemic lupus erythematosus (SLE) and Mixed Connective Tissue Disease ILD (MCTD), are associated with ILD [30–33]. There has been a high but divergent reported incidence of ILD among patients with SSc, ranging from 40 to 91% [34, 35]. According to several studies, the prevalence of pulmonary involvement in SS varies widely from 9 to 26% [36, 37]. Based on HRCT and PFT findings, ILD is reported in 47 to 90% of patients with MCTD, whereas ILD is reported in 1 to 15% of patients with SLE [38]. Although PF remains a rare disease, statistics show that the mortality rate is steadily increasing (from 18.81 per 100,000 people in 2000 to 20.66 per 100,000 people in 2017) [39, 40]. A rising mortality rate indicates that PF remains a pressing challenge and that a deeper understanding of its pathogenesis is necessary. In addition to having an innate immune response, NETs may direct the progression and occurrence of fibrosis, making them a new target for anti-fibrotic therapies.

### NETs induce pulmonary fibrosis through fibroblasts

Fibroblasts that form the lung connective tissue are derived mainly from embryonic mesenchymal cells. Lung fibroblasts, as the main constituent cells of lung connective tissue, are postulated to perform the following functions under physiological conditions: maintain the normal shape of lung tissue, provide the scaffold to facilitate effective gas exchange of lung tissue, and synthesize and secrete a variety of proteins, collagen fibers and other extracellular matrix components after lung tissue damage has been rapidly repaired [41–43].Under pathological conditions, inflammation of the alveolar epithelial cells and sustained damage cause the recruitment of large numbers of immune cells, which then produce and release pro-inflammatory and pro-fibrotic factors [44, 45]. As a result of the combined action of various cytokines, lung fibroblasts are abnormally activated. Activated lung fibroblasts are the key effector cells of PF. They can, for example, contribute directly to the occurrence and development of PF through abnormal proliferation, phenotypic transformation, and secretion of extracellular matrix (ECM) components [46–48]. Moreover, lung fibroblasts can indirectly promote fibrosis by secreting inflammatory factors such as Interleukin-1(IL-1), IL-6, and tumor necrosis factor-alpha (TNF-α) [49].

NETs play a crucial role in the activation of lung fibroblasts and their differentiation into myofibroblasts. Effects of NETs components MPO and histones on the proliferation and differentiation of lung fibroblast were confirmed by MPO inhibitor or histones inhibitor with the down-regulation of alpha-smooth muscle actin(α-SMA) and collagen production. Whereas pre-treated with recombinant human MPO (rhMPO) and rhhistones3 promoted the expression of α-SMA, the collagen production and the proliferation of lung fibroblasts. And the DNA components of NETs was confirmed by Toll-like receptor 9(TLR9)-miR-7-drosophila mothers against decapentaplegic 2(Smad2) signaling pathway to promote lung fibroblast proliferation and their differentiation into myofibroblasts [50]. In vitro activation and differentiation of human lung fibroblasts into myofibroblasts by NETs released from neutrophils treated with phorbol 12-myristate 13-acetate (PMA) resulted in elevated mRNA levels for a disintegrin and metalloprotease 12 (ADAM12), actin alpha 2 (ACTA2) and protein levels for the mesenchymal marker α-SMA and collagen production. However, the activity of NETs on lung fibroblasts after digestion with DNase was eliminated [51], indicating that DNA components in NETs likely influence the differentiation of fibroblasts positively. Despite not directly stimulating fibrotic responses on fibroblasts in vitro, the fibrosis-inducing drugs, cigarette smoke extract (CSE), magnesium silicate and bleomycin indirectly induce fibroblast activation through NETs release [51]. Furthermore, these investigators found that the expression of IL-17 in NETs components did not affect the differentiation of lung fibroblasts into myofibroblasts, but IL-17 acted in concert with DNA/histones in NET-dependent fibroblast activation. The derived components of NETs produced under different conditions vary, and although they do not directly determine the activated differentiation of fibroblasts, they can indirectly participate in their fibrogenesis process [51]. Studies showed that active SLE expresses tissue factor (TF)-bearing and IL-17A-bearing NETs, which activate and differentiate human skin fibroblasts. In elucidating the components of NETs responsible for activation and differentiation of human skin fibroblasts, TF and IL-17A were found, in particular, to decorate active SLE NETs. Researchers assessed the expression of ACAT2 and found that TF or IL-17A neutralization of NETs did not mediate human skin fibroblasts activation/differentiation. Collectively, these results demonstrate that active SLE NETs contribute to the activation/differentiation of human skin fibroblasts, whereas TF and IL-17A present in SLE NETs enhance the fibrotic activity of differentiated human skin fibroblasts [52]. Together, these findings indicate the presence of NET-derived components, suggesting their involvement in fibrotic aspects. Although some authors have demonstrated the effects of different components of NETs on lung fibroblast proliferation and differentiation, the mechanisms by which some components of NETs exert these effects remain unclear.

**Fig. 1:**
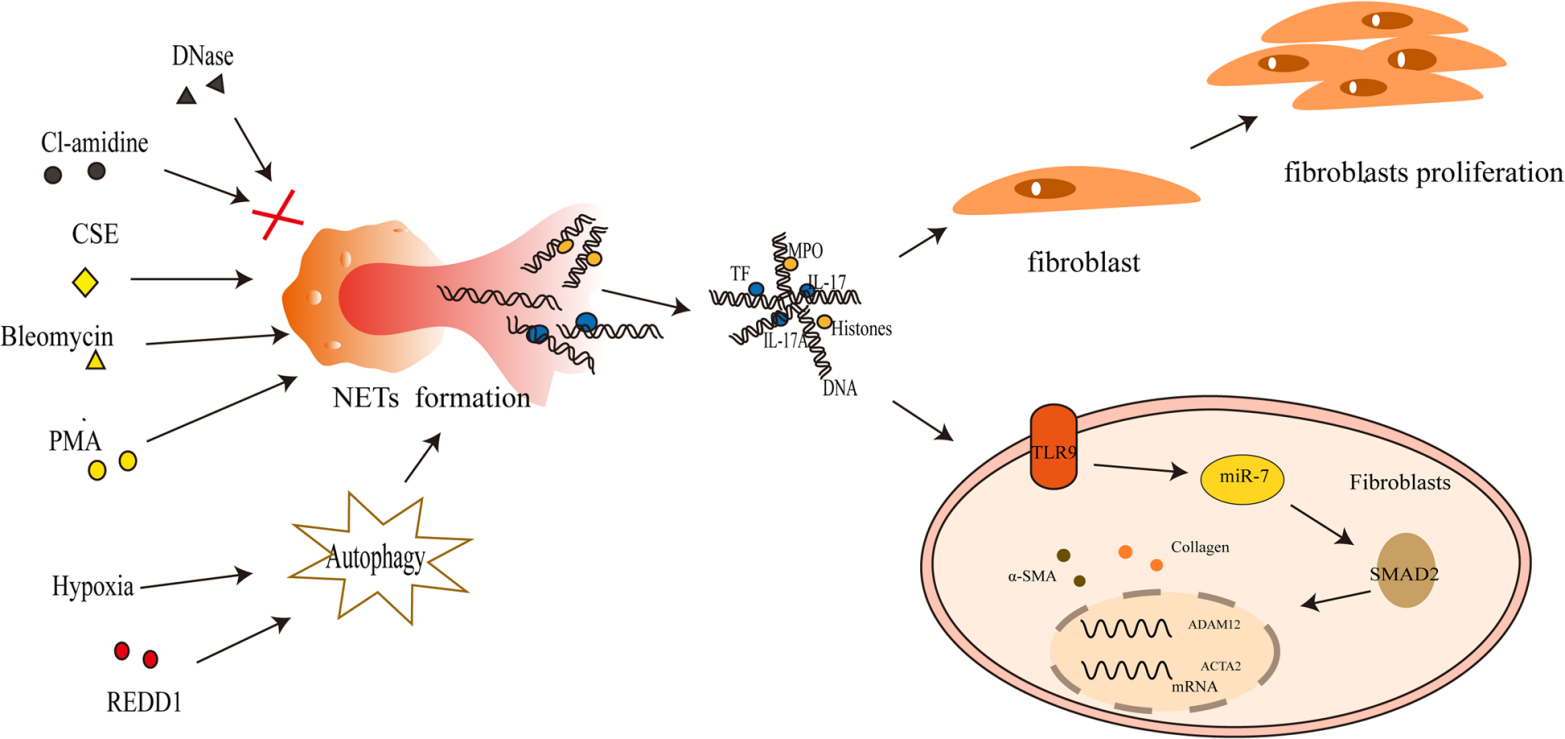
NETs induce pulmonary fibrosis through fibroblasts. PMA, Bleomycin, CSE could promote the release of NETs from neutrophils. Under hypoxia conditions, REDD1 promotes NETs release by enhancing autophagy, while Cl-amidine (a PAD4 inhibitor) and DNase prevent NETs production and action process. It has been suggested that MPO and Histones contribute to the proliferation and differentiation of fibroblasts, whereas DNA promotes myofibroblast differentiation by connecting with the pathway TLR9-miR-7-SMAD2. There is evidence that IL-17, IL17A, and TF promote fibrotic activity in differentiated fibroblasts, but not their differentiation. NETs: neutrophil extracellular traps; PMA: phorbol 12-myristate 13-acetate; CSE: cigarette smoke extract; PAD4: peptidylarginine deiminase type 4; MPO: myeloperoxidase

PAD4 also promotes the release of NETs through histone guanylation [24]. Inducing PF in PAD4-KO mice with bleomycin suppressed the expression of fibrosis-associated mediators significantly, including collagen type I α 1 chain, elastin, fibronectin 1, connective tissue growth factor and fibroblast growth factor 2 [53]. The PAD4 deficiency prevented a decrease in alveolar epithelial and pulmonary vascular endothelial cell numbers and increased ACTA2-positive mesenchymal cells and S100A4-positive fibroblasts in the lung. Pretreatment of neutrophils with Cl-amidine, a PAD4 inhibitor that inhibits NETs release by blocking guanylation of histone H3 and subsequent incubation with cigarette smoke extract inhibited the differentiation and activation of fibroblasts [53]. The findings suggest that the PAD4 inhibitor is a therapeutic target for PF because it reduces bleomycin-induced NETs and fibrosis formation. This promising research area will require further investigation into how anti-NETs interrupt PF and represents a potential treatment (Fig. 1).

### NETs drive lung epithelial injury and pulmonary fibrosis

Repetitive injury and alterations of the alveolar epithelium (including alveolar epithelial cell proliferation and hyperplasia) [54], abnormal alveolar epithelial cell activation that leads to impaired epithelial-mesenchymal crosstalk [55] and senescence of pulmonary epithelial cells [56] have been described and linked with the development of PF. Currently, PF is postulated to be caused by repeated damage to alveolar epithelial cells, which can cause abnormal interactions between epithelial cells and fibroblasts [57, 58]. Cell death of alveolar epithelial may occur during the early stages of PF [59, 60], in which alveolar epithelial cell type II (ATII) damage is significant, and there have been reports that in the lungs of patients with IPF, 70–80% of the ATII stained positive for apoptotic markers [61, 62].

NETs play various roles in lung epithelial injury and fibrosis. For example, protein components of NETs, particularly histones, caused epithelial and endothelial cell death in a mouse model of acute lung injury induced by lipopolysaccharide [63]. In vitro, extracellular histones are cytotoxic to endothelial cells, while in vivo, histone administration leads to neutrophil margins, vacuolar endothelial cells, intra-alveolar hemorrhage, and macro- and microvascular thrombosis [64]. Other pathways besides NETs-DNA may also damage lung epithelial cells, such as NETs mediating inflammasome activation and IL-1β secretion from monocytes and causing airway epithelial cell injury, and NETs modify and regulate lung injury by activating ferroptosis in alveolar epithelial [65–67]. Additionally, NETs are associated with the development of lung fibrosis after COVID-19, because NETs induce the downregulation of the epithelial marker E-cadherin and the upregulation of the mesenchymal marker α-SMA in pulmonary epithelial cells [68–71]. Exposure to butyl benzyl phthalate (BBP), a widely used plasticizer, has been linked to asthma and impaired lung functions [72, 73]. As a consequence of promoting glucose uptake and ROS burst in neutrophils, BBP promotes EMT through the formation of NETs [74]. The regulation of NETs on lung epithelial cells is primarily determined by epithelial cell injury and mesenchymal transition, and there remains much to be learned about the role of NETs in driving pulmonary epithelial injury and PF.

### NETs-mediated inflammation and pulmonary fibrosis

PF is commonly associated with chronic inflammation. A complex network of cytokines, chemokines and inflammatory mediums are released during the process of PF, driving fibroblast recruitment, proliferation and overproduction of ECM. Key cytokines and chemokines that induce a pro-fibrotic environment include TNF-α, transforming growth factor-β (TGF-β), monocyte chemotactic protein 1 (MCP1)/CCL2, macrophage inhibitory protein 1α (MIP1α)/ CCL3 and T-2-chemokines such as CCL17, CCL18 and CCL22 [75, 76]. TGF-β is an important mediator of pro-fibrosis and induces fibroblast differentiation into myofibroblasts. TGF-β also promotes ECM production by promoting ECM gene transcription (including collagen, fibronectin and proteoglycan) and preventing collagen degradation by inhibiting matrix metalloproteinase, fibrinogen activator and elastase activities [58, 77, 78]. NE is a main protein component of NETs and an inhibitor of NE has been demonstrated to attenuate PF in a murine model by inhibiting TGF-β1 and inflammatory cell recruitment to the lungs [79]. The mechanism by which NETs rely on TGF-β as a signaling pathway has not been investigated in detail; however, NETs have been shown to activate TGF-β in other diseases. For example, NETs induce proliferation, invasion, migration, and EMT of gastric cancer cells by activating the TGF-β-p-Smad2/3 signaling pathway [80]. NETs are associated with the upregulation of the TGF-β signaling pathway and associated genes, including TGF-β1, Smad3 and collagen type III α 1 chain in chronic thromboembolic pulmonary hypertension monocytes. Further studies have indicated that NETs-dependent monocyte differentiation causes a predominantly fibroblast phenotype with increased TGF-β-dependent signal transduction [2]. TGF-β may act as a downstream molecule of NETs to promote the progression of PF. Moreover, NETs may promote the release of TGF-β, an important agent for tissue remodeling after acute lung injury during late phases [81]. According to existing studies, NETs probably activate the TGF-β signaling pathway in lung fibroblasts to promote PF progression. Confirmation of this activation process requires further research efforts.

**Fig. 2:**
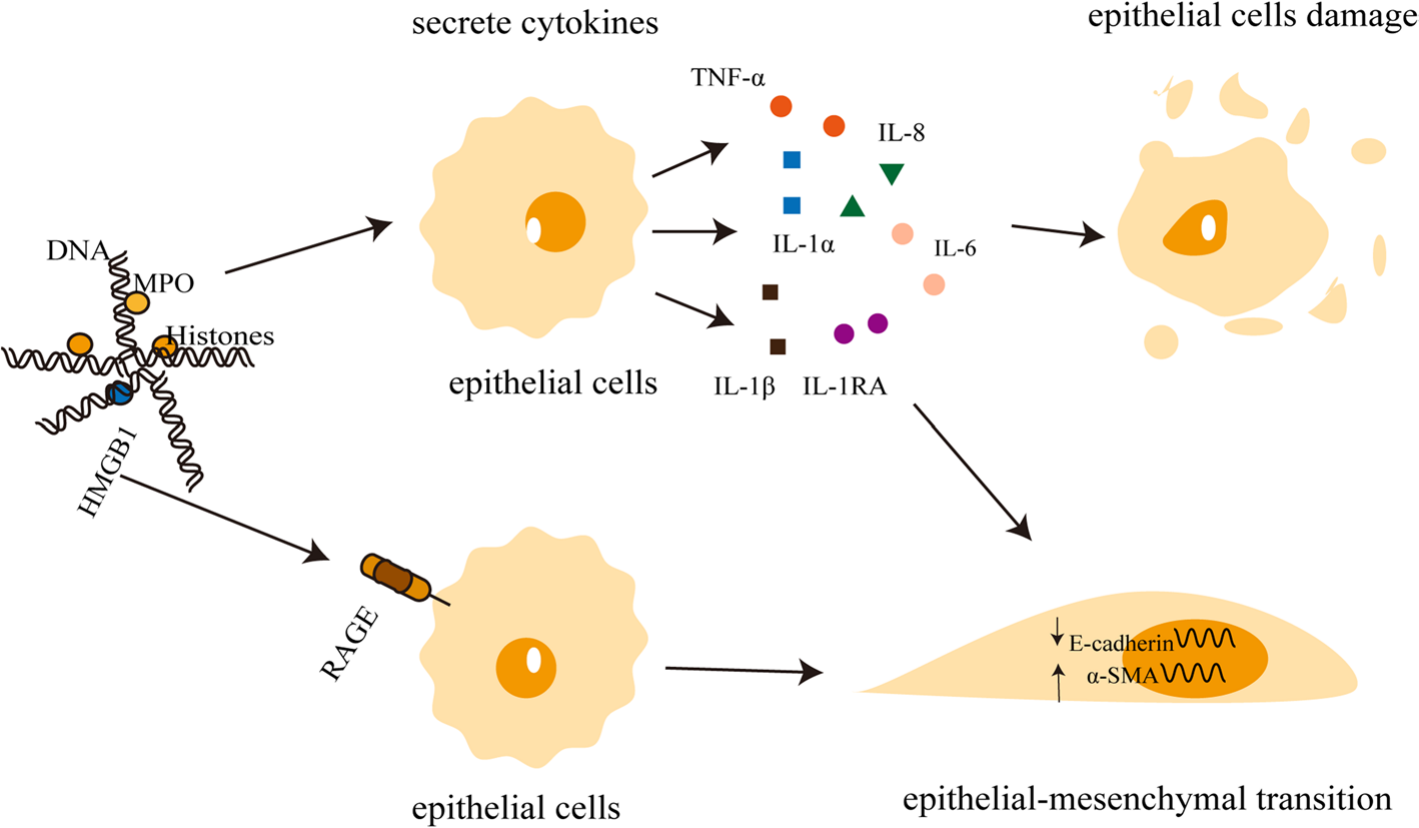
NETs drive lung epithelial injury and pulmonary fibrosis. MPO, DNA and histones in Nets can cause damage to alveolar epithelial cells, which release a large number of pro-inflammatory and pro-fibrotic factors (including IL-6, IL-8, IL-1α, IL-1β and TNF-α) and initiate subsequent inflammatory immune repair. Excessive repair of alveolar epithelial cells after injury is an important mechanism of pulmonary fibrosis. HMGB1 in NETs could act on the RAGE receptors in lung epithelial cells, which in turn leads to the downregulation of the epithelial cell marker E-cadherin and the upregulation of the myofibroblast cell marker α-SMA. NETs: neutrophil extracellular traps; MPO: myeloperoxidase; HMGB1: high mobility group box 1; RAGE: the receptor of advanced glycation end-product; α-SMA: alpha-smooth muscle actin

NETs also stimulate the alveolar and bronchial epithelial cells to secrete pro-inflammatory factors such as IL-8 and IL-6, not by inducing DNA or histones in the cells, but by releasing the high mobility group box 1 (HMGB1) protein. HMGB1 induces the secretion of IL-8 and IL-6 via the receptor of advanced glycation end-product (RAGE) [82]. Studies have shown that the mRNA of RAGE is highly expressed in alveolar epithelial cells and the nuclear protein HMGB-1 was also identified in NETs [83]. HMGB-1 was also shown to induce the release of pro-inflammatory cytokines when released into the extracellular environment [84–86]. IL-17A acts as a pro-inflammatory factor that promotes PF by depending on NETs [51, 87]. Furthermore, NETs also promote the release of cytokines such as IL-1, TNF-α, IL-1RA and IL-1α [51, 88, 89]. The essential role of NETs-produced inflammatory factors is clearly important in PF progression. Interestingly, Chirivi and his colleagues described the inhibition of NETs with the therapeutic anti-citrullinated protein antibody (tACPA) for the first time in mice models. By using tACPA, it may be possible to reduce neutrophil-driven inflammation and decrease lung fibrosis [90]. According to the evidence presented above, NETs cause chronic lung inflammation by producing a series of inflammatory factors that, in turn, stimulate fibrotic pathways downstream in cells (such as lung epithelial cells, fibroblasts and immune cells) to accelerate the progression of PF (Fig. 2).

### Autophagy-driven NETs and pulmonary fibrosis

Autophagy is a self-degradative process responsible for balancing energy sources during critical developmental stages and in response to nutrient stress [91]. Rather than eliminating materials, autophagy serves as a dynamic recycling system that creates new building blocks and energy to maintain the integrity of cells and homeostasis. Abnormalities in the autophagic process may cause the development of diseases [92]. Autophagy is involved in the NETosis process, and different types of vesicles with a double phospholipid bilayer are found in PMA-stimulated neutrophils and monitoring the localization of the autophagy marker microtubule-associated protein 1 light chain 3 suggests that PMA induces autophagy in a superoxide-independent manner. Additionally, the pharmacological inhibition of autophagy did not affect the activity of PMA-induced NADPH oxidase [93]. A significant reduction in the release of NETs was observed after treating neutrophils with the autophagy inhibitors bafilomycin A1 and chloroquine [51, 94]. Typically, autophagy is a protective mechanism for an organism, with enhanced autophagy reducing the progression of PF [95–99]. In contrast excess of autophagy stimulates the activation of fibroblasts and causes tissue fibrosis. For example, the coactivator peroxisome proliferator-activated receptor gamma coactivator-1α is upregulated in SSc patients and mouse animal models to promote autophagy for TGF-β-induced fibroblasts activation [100]. TGF-β also activates autophagy through epigenetic mechanisms to amplify its pro-fibrotic effects [101]. The hypoxia-response and stress-response protein (REDD1)/ autophagy/ NETs axis is involved in kidney injury and dermatofibrosis in patients with SLE. Studies showed that endothelin-1 and hypoxia-inducible factor-1α inhibition, prior to stimulation of neutrophils with an active serum, ameliorated the activation/differentiation of human skin fibroblasts to myofibroblasts, indicating the importance of NETs in the activation/differentiation of fibroblasts [52]. Additionally, the REDD1/autophagy pathway has been reported to induce NETs and increase IL-1β secretion [102]. Although the regulatory mechanism of PF by autophagy/NETs has not been fully resolved, we were able to detect a possible role for autophagy/NETs in PF regulation.

## Conclusion

NETs are associated with the early and advanced stages of PF. Evidence suggests that NETs may regulate the fibrosis process by causing pro-inflammatory effects, injuring pulmonary epithelium and endothelium, promoting pulmonary EMT, activating lung fibroblasts, or inducing autophagy. Further research is required to fully understand how NETs interact with cells and downstream signaling pathways during lung fibrosis. Current research on NETs and PF is based primarily on basic experiments, and potential clinical transformation still requires further exploration. Signaling pathways and molecular targets related to NETs have been partially identified, so inhibiting NETs to delay disease progression or onset should still be possible.

## Data Availability

Not applicable.

## Abbreviations

PF: Pulmonary fibrosis
NETs: Neutrophil extracellular traps
MPO: Myeloperoxidase
NE: Neutrophil elastase
EMT: Epithelial-mesenchymal transformation
ROS: Reactive oxygen species
PAD4: Peptidylarginine deiminase type 4
ILD: Interstitial lung disease
IPF: Idiopathic pulmonary fibrosis
CTDs: Connective tissue diseases
SSc: Systemic sclerosis
SS: Sjögren’s syndrome
SLE: Systemic lupus erythematosus
MCTD: Mixed Connective Tissue Disease ILD
ECM: Extracellular matrix
IL-1: Interleukin-1
TNF-α: Tumor necrosis factor alpha
rhMPO: Recombinant human MPO
TLR9: Toll-like receptor9
PMA: Phorbol 12-myristate 13-acetate
ADAM12: A disintegrin and metalloprotease 12
ACTA2: Actin alpha 2
TF: Tissue factor
α-SMA: Alpha-smooth muscle actin
CSE: Cigarette smoke extract
ATII: Alveolar epithelial cell type II
Smad2: Drosophila mothers against decapentaplegic2
BBP: Butyl benzyl phthalate
TGF-β: Transforming growth factor-β
MCP1/CCL2: Monocyte chemotactic protein 1
MIP1α/ CCL3: Macrophage inhibitory protein 1α
HMGB1: High mobility group box 1
tACPA: Therapeutic anti-citrullinated protein antibody
REDD1: The hypoxia-response and stress-response
RAGE: The receptor of advanced glycation end-product
